# Hemorrhagic Fever Disease in STAT-1 Knockout Mice Infected with Lujo Virus

**DOI:** 10.3390/pathogens15040394

**Published:** 2026-04-07

**Authors:** Dylan M. Johnson, Sharon Jan, Ethan Dunn, Jason E. Comer, Robert W. Cross, Thomas W. Geisbert

**Affiliations:** 1 Texas Biomedical Research Institute, San Antonio, TX 78227, USA; 2 Sandia National Laboratories, Livermore, CA 94550, USA; 3 Galveston National Laboratory, Department of Microbiology and Immunology, University of Texas Medical Branch, Galveston, TX 77555, USA

**Keywords:** Lujo virus, Lujo hemorrhagic fever, arenavirus, viral hemorrhagic fever, viral pathogenesis, medical countermeasure development, animal models of infection

## Abstract

Lujo virus (LUJV) is an arenavirus that causes Lujo Hemorrhagic Fever (LHF), a viral hemorrhagic fever that emerged in a 2007 outbreak in Zambia and South Africa with an 80% case fatality rate and evidence of human-to-human nosocomial transmission. There are no approved medical countermeasures for LHF, although several screens have identified lead antiviral compounds. The lack of accessible animal models limits the development of lead compounds and characterization of broadly protective anti-arenavirus compounds such as ribavirin for the treatment of LHF. Here, we present preliminary data characterizing the partially lethal disease caused by LUVJ in STAT-1 deficient mice. Several key hematological, clinical chemistry, and histologic findings common to LHF disease are recapitulated in this model. This work suggests that further characterization of LUJV infection in STAT-1 deficient mice may allow development of a model that would be instrumental in the development of medical countermeasures for LHF.

## 1. Introduction

*Lujo mammarenavirus* (LUJV) first appeared in an infected patient in the Lusaka region of Zambia in 2008. The patient was transferred to Johannesburg, South Africa for treatment which initiated a nosocomial outbreak of Lujo hemorrhagic fever (LHF), a viral hemorrhagic fever disease with an unusually high (4/5, 80%) case fatality rate [1,2]. LHF in humans is associated with acute respiratory distress, impaired renal function, thrombocytopenia, leukopenia, granulocytopenia, hepatocyte necrosis, and elevated (alanine and aspartate) transaminase levels [2]. Ribavirin, a broad-spectrum antiviral, was used as treatment for two patients, with one patient receiving an earlier course of both oral followed by intravenous treatment surviving LHF [2]. The use of ribavirin has been recommended for LHF; however, concerns of exacerbation of viral hemorrhagic fever diseases due to ribavirin’s renal and hepatic toxicity, along with the appropriate treatment window, remain a topic of debate [3,4,5,6].

LUJV is most closely related to other Old World arenaviruses, including *Lassa mammarenavirus* (LASV), the causative agent of Lassa Fever (LF), and Lymphocytic Choriomeningitis virus (LCMV), associated with disease in immunocompromised patients, as well as numerous other non-pathogenic African arenaviruses including *Mopeia mammarenavirus* (MOPV), *Ippy mammarenavirus* (IPPV), and *Mobala mammarenavirus* (MOBV) [7]. Although the LUJV glycoprotein cross reacts with LASV, LCMV, and MOBV, but not MOPV or IPPV immune sera [8], the entry receptor for LUJV appears unique among arenaviruses [9,10,11]. Despite surveillance efforts in Zambia, the (presumed rodent) reservoir remains unidentified [12,13,14].

LHF has been modeled in strain 13 guinea pigs with features that align to human disease. However, there are challenges with strain 13 models of arenavirus infection including the lack of commercially available strain 13 guinea pigs, limited immunological reagents for guinea pigs in general, and limited genetic information about this strain [15]. Additionally, strain 13 guinea pigs can show hemorrhagic fever disease signs when infected with mammarenaviruses that are thought to be apathogenic in humans [16]. LUJV infection of cynomolgus macaques resulted in a mild illness characterized by delayed inflammatory and apoptotic host responses [17]. STAT-1 constitutive knockout mice (STAT-1^−/−^) [18,19,20,21,22], interferon alpha receptor knockout [23,24] and interferon alpha receptor and interferon gamma receptor double knockout mice (IFrag^−/−^) [23,25,26] have been described as murine models of other arenavirus hemorrhagic fever diseases but have yet to be used in characterizing LUJV. The exploratory hypothesis of this research is that because immunocompromised mouse models were described for other arenaviruses, they may also be instrumental to the aim of developing a murine model of a LHF-like disease. Because of prior successes with model development for other arenavirus hemorrhagic fevers in STAT-1^−/−^ and interferon deficient mice, these model systems were selected for preliminary study.

Murine models of arenavirus disease are instrumental in medical countermeasure development [27]. Medical countermeasure development for LHF has likely been hindered by the lack of accessible small animal models of disease. No vaccines have been developed for LHF [28], although the application of a LUJV reverse genetics reporter system [29] was key in the identification of several lead compounds for LHF drug development [30,31]. Here, we present data from LUJV infection of STAT-1^−/−^ and IFrag^−/−^ mice providing preliminary evidence of their potential utility as models of LUJV infection that could be used for the in vivo development of LHF medical countermeasures.

## 2. Materials and Methods

Four- to six-week-old STAT-1^−/−^ mice (129S6/SvEv-Stat1^tm1Rds^, *n* = 10, 5 males and 5 females) were purchased from Taconic (Germantown, New York, NY, USA). IFrag^−/−^ mice (B6.Cg-Ifngr1^tm1Agt^ Ifnar1^tm1.2Ees^/J, *n* = 5, 3 males, 2 females, 65 days-old on the day of virus inoculation) were purchased from the Jackson Laboratory (Bar Harbor, ME, USA). Mice were housed at the University of Texas Medical Branch (UTMB) Galveston National Laboratory under ABSL4 conditions with up to 5 mice per cage segregated by strain and sex. Mice were housed in sterile Techniplast Green Line caging (West Chester, PA, USA) with corncob bedding, and shredded foraging paper and cardboard huts for enrichment. STAT-1^−/−^ mice were acclimated to BSL4 for 4 days prior to inoculation; IFrag^−/−^ were acclimated for 9 days. Implantable electronic identification and temperature transponders (Bio Medic Data Systems, Seaford, DE, USA) were implanted subcutaneously under isoflurane anesthetic the day prior to virus inoculation for STAT-1^−/−^ mice and 5 days prior to virus inoculation for IFrag^−/−^ mice. All mice were allocated to the LUJV challenge group and were inoculated intraperitoneally under isoflurane anesthetic on study day zero with 9.0 × 10^3^ plaque forming units (PFU) of LUJV strain Prototype Zambia (200809232). A target dose of 1.0 × 10^4^ was selected based on previously published work with LASV [18]. The actual challenge dose of 9.0 × 10^3^ was confirmed via plaque assay of the infectious inoculum on Vero 76 cells under 0.8% agarose overlay with the addition of neutral red 5 days following infection and plaque counting the following day, as previously described [32]. This study design precluded randomization and blinding, and would not be expected to be robust to cage effect bias. Mice were weighed and clinically scored daily following inoculation and were euthanized when they met humane endpoint criteria or at 28 days post-inoculation (DPI). All animal procedures were approved by the UTMB Institutional Animal Care and Use (IACUC) and Institutional Biosafety (IBC) committees.

At the time of euthanasia, blood was collected into MiniCollect K2EDTA plasma tubes (Greiner Bio-One, Monroe, NC, USA) and analyzed on a Vetscan HM5 (Zoetis, Parsippany, NJ, USA). Remaining blood was centrifuged and plasma was collected for analysis with a Piccolo BioChemistry Panel Plus (Abbott Laboratories, Abbott Park, IL, USA). Historical controls (HC) were included from a previously published study of STAT-1^−/−^ mice which were processed similarly except a Vetscan VS2 Comprehensive Diagnostic Profile was used for clinical chemistry measurements [33].

Liver, spleen, kidney, lung, and brain tissues were collected and preserved by RNAlater (Thermo Fisher Scientific, Waltham, MA, USA) for quantitative reverse transcriptase PCR (qRT-PCR) viral load quantification, or in 10% neutral buffered formalin (Thermo Fisher Scientific, Waltham, MA, USA) for histology. RNA was isolated from RNAlater using an RNEasy kit (Qiagen, Hilden, Germany). Inactivated RNA was removed from the BSL4 according to UTMB IBC approved procedures. Quantitative reverse transcriptase PCR (qRT-PCR) was conducted on a CFX96 Real-Time PCR Detection System (Bio-Rad Laboratories, Berkeley, CA, USA) using a QuantiNova Probe RT-PCR kit (Qiagen, Hilden, Germany) with the following primer and probe set: Forward—5′-CTTTC GTTCT GTGGA GGTGT AG-3′; Reverse—5′-CATCT CTTCC AGAAC CAGCA TAA-3′; Probe—5′-6-FAM-CTGTG TTGT-ZEN-CCCAG GATCT GCCTT-3′-IABkFQ (Integrated DNA Technologies, Coralville, IA, USA). Results were normalized to a mouse β-actin primer-probe set (cat# Mm.PT.39a.22214843.g, Integrated DNA Technologies, Coralville, IA, USA) and quantified based on comparison to a standard curve generated with a synthetic DNA target: ctcac accca cagga aattg gtggg gggct tttgc tcttt cgttc tgtgg aggtg taggg tcctt tggag tcatat caatg actgt gttgt cccag gatct gcctt ttatg ctggt tctgg aagag atgta tggcc (Integrated DNA Technologies, Coralville, IA, USA).

Formalin fixed tissue was removed from the BSL4 according to University of Texas Medical Branch (UTMB) institutional biosafety committee approved procedures, embedded in paraffin, sectioned, stained with hematoxylin and eosin, and imaged on a Zeiss Axioscan slide scanner (Oberkochen, Germany).

Figures were made with Prism (version 10, GraphPad Software, Boston, MA, USA) with specific statistical analysis reported in the figure legends. Single mice were treated as individual experimental units (data points) for analysis. No data were excluded from analysis except when insufficient sample volume for clinical chemistry led to a single LUJV infected IFrag^−/−^ being excluded from this analysis; and one LUJV infected STAT-1^−/−^ did not return an alanine aminotransferase value due to sample hemolysis.

## 3. Results

**Figure 1:**
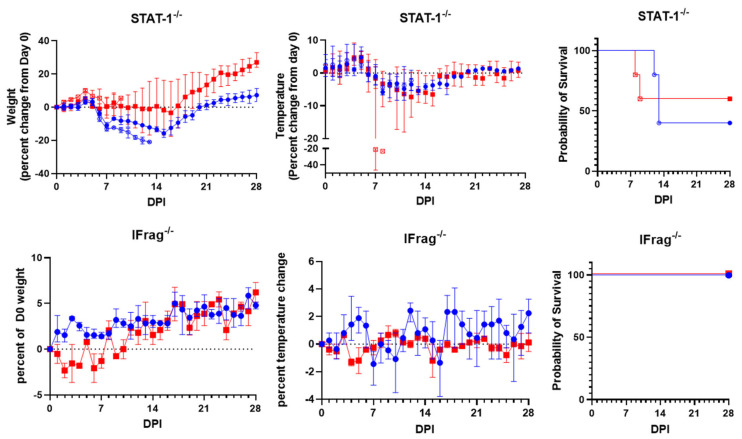
STAT-1^−/−^ (**top**, *n* = 10, 5 males [blue circles], 5 females [red squares]) and IFrag^−/−^ (**bottom**, *n* = 5, 3 males [blue circles], 2 females [red squares]) were challenged with 9.0 × 10^3^ PFU of LUJV and monitored daily for weight (**left**), temperature (**middle**), and survival (**right**). Weights and temperatures are reported as the percent change from 0 days post-inoculation (DPI). STAT-1^−/−^ subjects that met euthanasia criteria prior to the planned end of the study are graphed separately for weight and temperature and indicated with open symbols. Error bars represent the range of measurements for each study day.

STAT-1^−/−^ mice had progressive weight loss following LUJV inoculation starting five days post-infection (DPI). Survivors began to gain weight at about two weeks post-infection (Figure 1 top left). A fever was observed at 4 and 5 DPI in most subjects followed by hypothermia, with severe hypothermia observed in the final measurement from both female subjects meeting early euthanasia criteria (Figure 1 top middle). Half of the animals (5 of 10) met early euthanasia criteria by 13 days post-LUJV inoculation (Figure 1 top right). Three mice met early euthanasia criteria due to loss of more than 20% of their initial body weight. The other two meeting early euthanasia criteria both had severe hypothermia with one displaying ataxia and the loss of righting reflex, while the other was moribund. Of these, gross anatomical observation revealed necrotic pneumonia in 2 of 5 subjects, splenomegaly in 3 of 5 subjects, and a liver pallor in 1 of 5 subjects. No significant lesions were observed during the necropsy of STAT-1^−/−^ mice surviving to the planned end of the study at 28 DPI.

IFrag^−/−^ mice had transient weight loss lasting about a week followed by normal weight gain after LUJV infection (Figure 1 bottom left). Body temperatures varied throughout the experiment but did not show a detectable disease related pattern (Figure 1 bottom middle). All IFrag^−/−^ mice survived until the planned end of the study 28 DPI (Figure 1 bottom right). Both female IFrag^−/−^ had necrotic lesions on the distal spleen at necropsy, but otherwise no significant lesions were noted during necropsy.

**Figure 2:**
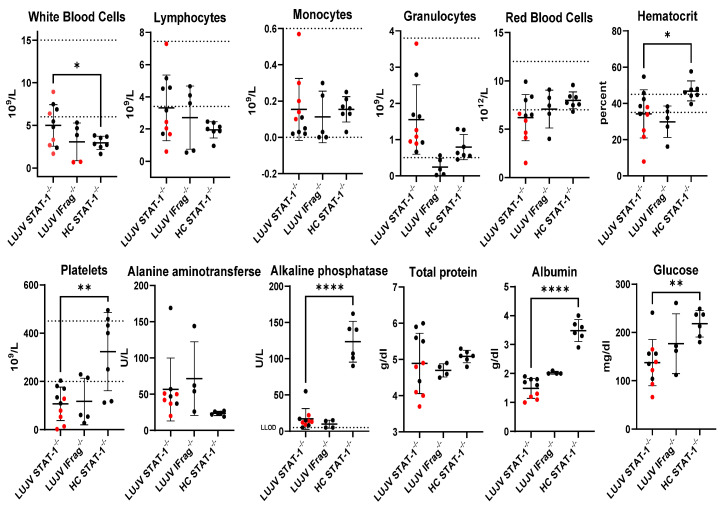
Hematology and clinical chemistry values prior to euthanasia. Each closed circle symbol represents data from a single mouse. Data points in red represent subjects that met early euthanasia criteria prior to the planned end of the study. Dashed lines on hematology values represent the typical normal values for healthy mice provided by the manufacturer of the HM5 analyzer. The dashed line on alkaline phosphatase represents the lower limit of detection (LLOD) of 5 U/L; 2 samples each from LUJV infected STAT-1^−/−^ and IFrag^−/−^ mice were below the LLOD and are plotted at the value of 5 U/L. Error bars denote the mean value with standard deviation. Insufficient sample volume for clinical chemistry led to a single LUJV infected IFrag^−/−^ being excluded from this analysis. One LUJV infected STAT-1^−/−^ did not return an alanine aminotransferase value due to sample hemolysis. An unpaired Student’s *t*-test was used to compare LUJV infected STAT-1^−/−^ to uninfected historical control (HC) STAT-1^−/−^ mice from a prior study [33], * *p* < 0.05; ** *p* < 0.01; **** *p* < 0.0001.

Hematologic values showed a trend towards leukocytosis, anemia, and thrombocytopenia consistent with viral hemorrhagic fever disease (Figure 2). Significantly elevated alanine transaminase levels suggest hepatic dysfunction, a typical finding during arenavirus hemorrhagic fever disease (Figure 2). Low albumin may suggest that subjects experienced systemic vascular failure and/or renal dysfunction as well (Figure 2).

**Figure 3:**
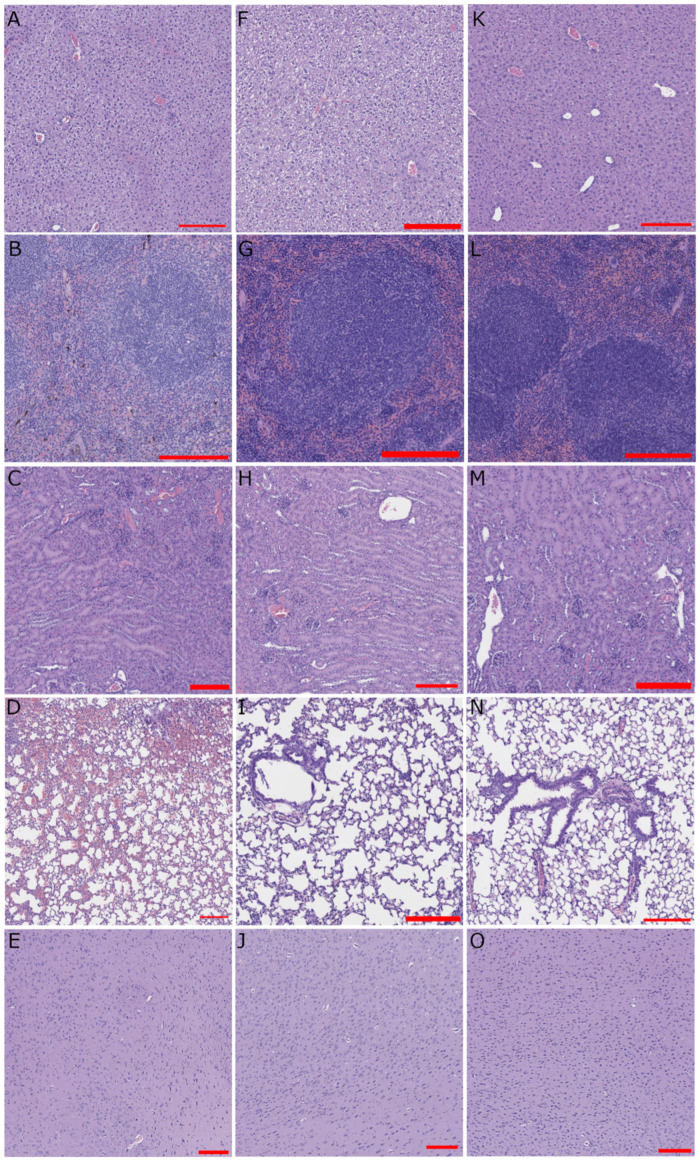
Histopathology. Representative images from (**A**–**E**) a STAT-1^−/−^ LUJV infected mouse that met early euthanasia criteria, (**F**–**J**) a STAT-1^−/−^ LUJV infected mouse that survived until the planned end of the study, and (**K**–**O**) an IFrag^−/−^ LUJV infected mouse. (**A**,**F**,**K**)—liver; (**B**,**G**,**L**)—spleen; (**C**,**H**,**M**)—kidney; (**D**,**I**,**N**)—lung; (**E**,**J**,**O**)—brain. Red scale bars are 200 μm long.

Liver histology revealed diffuse hepatocellular degeneration and necrosis during LUJV infection of STAT-1^−/−^ mice in both subjects meeting early euthanasia criteria (Figure 3A) and those surviving to the planned end of the study at 28 DPI (Figure 3F). Splenic sections from most mice examined had some degree of splenic hemorrhage and parenchymal necrosis (Figure 3B,G,L). Renal infiltrates were observed in STAT-1^−/−^ mice during acute LUJV infection (Figure 3C) and in IFrag^−/−^ mice (Figure 3M). Pulmonary hemorrhage was apparent in STAT-1^−/−^ mice during acute LUJV infection (Figure 3D). Cortex sections appeared normal (Figure 3E,J,O). Overall, lung pathology was a differentiating feature between STAT-1^−/−^ mice that survived compared to those that met early euthanasia criteria.

Viral RNA was detected at relatively high levels in kidney, liver, and lung tissue of both STAT-1^−/−^ and IFrag^−/−^ mice surviving to the planned end of the study, with an interesting trend towards the highest viral load being detected in the lung. Markedly less RNA was present in the brain of these subjects. STAT-1^−/−^ mice that succumbed to LUJV infection tended to have a lower viral load present in tissues, with kidney having the most viral load and brain having the least.

## 4. Discussion

**Figure 4:**
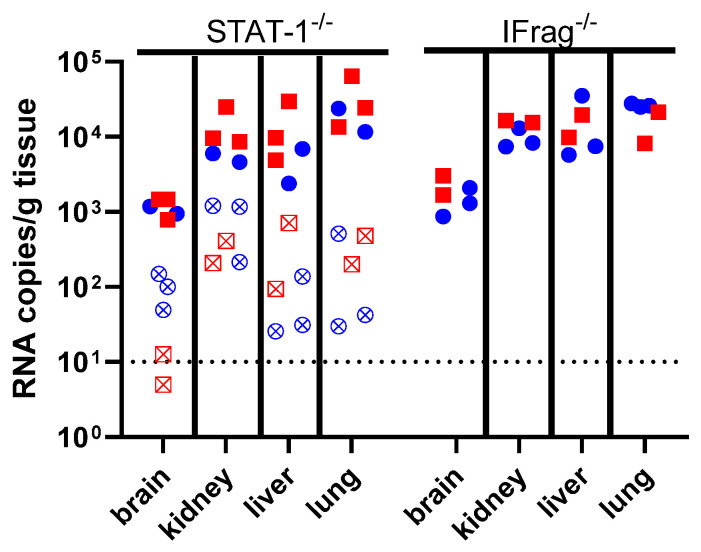
Viral quantification in tissues by qRT-PCR. LUJV RNA copies per gram (g) of tissue were quantified with a probe targeting the LUJV nucleoprotein gene. Blue circles represent male mice, and red squares represent female mice. Open symbols with an X are from subjects that met early euthanasia criteria. A dotted line at 10 RNA copies is the lower limit of quantification for the assay.

LUJV productively infected STAT-1^−/−^ mice causing lethal disease in half of the infected cohort. The disease caused a febrile illness followed by a period of weight loss and hypothermia (Figure 1). Mice that succumbed to LUJV infection had several hallmarks associated with LHF including low hematocrit, thrombocytopenia, and signs of viral hepatitis (Figure 2, Figure 3 and Figure 4). Interestingly, although STAT-1^−/−^ mice are generally susceptible to arenavirus disease, the pathogenicity of apathogenic-to-human arenaviruses [19,34], and partial lethality of human hemorrhagic fever causing arenaviruses [18,22] highlights that there is not a direct correlation between mortality in humans and STAT-1^−/−^ mice. Conversely, similarly to LASV infection of IFrag^−/−^ mice [23], although there are signs of LUJV viral replication in tissues (Figure 4), IFrag^−/−^ mice do not appear to show overt signs of infection.

LUJV causes severe illness in humans, and in the US it is a risk group 4 agent requiring handling under stringent biosafety level 4 containment. As a result, model development has been limited. This preliminary study utilized small group sizes (*n* = 10 for STAT-1^−/−^ mice and *n* = 5 for IFrag^−/−^ mice), which were limited by a combination of vendor availability coinciding with BSL4 schedule, which limits the generalizations that can be drawn from these data. However, these studies form the basis for work to fully characterize LUJV in STAT-1^−/−^ mice, allowing a better a priori estimate of sample size requirements for model refinement in the future. Age- and sex-related differences in LUJV pathogenesis were a feature of disease reported for strain 13 guinea pigs [35]. The small sample size limited the ability to reliably comment on sex-based differences in LUJV pathogenicity in this study.

Given the limited availability of both mice and available BSL4 schedule, a decision was made to use HC data. The decision to use HC data likely introduces cohort bias, and because of this, the hematology and clinical chemistry comparisons must be interpreted with care. However, the magnitude of significance despite the small sample size for many parameters, combined with the expected clinical profiles for arenavirus disease (typically including leukopenia, thrombocytopenia, and other hallmarks of viral hepatitis and hemorrhagic fever related systemic vascular failure) allow us to cautiously interpret these findings as significant. A more comprehensive study including a viral replication time course in tissues, with measurements of infectious virus by plaque assay, is a critical step to validate this model for antiviral therapy efficacy testing. It would also be preferable to describe the hematological and clinical chemistry outcomes compared to in-study controls, and future studies could also potentially include genetic background matched STAT-1 wild-type controls. Future studies could also be designed to probe the nature of the contribution of host responses to disease outcome.

In strain 13 guinea pigs, disease following LUJV appears to be heavily dependent on host immune responses [35]. This finding corroborates evidence that early immunosuppression in the non-human primate (NHP) model leads to mild illness in cynomolgus macaques [17]. In the case of LASV, the matrix protein Z and nucleoprotein antagonize host immunity (as recently reviewed [36]), but relatively little is known about the molecular pathogenicity and host interactions of LUJV. Mouse models are well-suited to the study of host responses to viral infection with many immunological tools available. Although, the immunocompromised nature of STAT-1^−/−^ mice can limit the generalization of immunological findings, this model could still be useful in targeted testing of LUJV induced host responses. Future studies probing these host responses could enable a better understanding of both LUJV molecular pathogenesis and allow insight into the mechanisms of action of antiviral therapies.

This represents the first preliminary description of a commonly available rodent model for LUJV infection. Because LUJV has not re-emerged since the initial outbreak, research funding for LUJV is limited. The two previously described models, although valuable for medical countermeasure development, come with accessibility challenges. Strain 13 guinea pigs are not commercially available and must be maintained in dedicated private breeding colonies. The NHP model of LUJV infection, while useful as an infection model, has limited signs of clinical illness. Limited accessibility of these models creates logistical hurdles making it difficult for first-in-animal screening of medical countermeasures.

Future work to expand the STAT-1^−/−^ mouse model of LHF, particularly increasing sample sizes, conducting dose-ranging experiments to determine if the partially lethal phenotype is related to infectious dose, determining viremia and infectious viral loads in tissues, localizing infection in tissues, and characterizing the host responses to infection are needed to expand this preliminary study. Furthermore, additional time points would allow an understanding of infection kinetics that could inform decisions on the appropriate time-to-treat during antiviral therapy testing. Ultimately, host response characterization would likely be insightful into the comparative molecular pathogenicity of arenaviruses.

Although LUJV is genetically related to LASV, it uses a different receptor from both LASV and the South American hemorrhagic fever causing arenaviruses. This divergence from other hemorrhagic fever arenaviruses, combined with its high lethality in humans makes LUJV an important outgroup for studying arenavirus viral pathogenesis and for testing antiviral compounds with broad arenavirus-family activity. Additionally, the wide availability of reagents and methodology to probe host immune responses in mice could facilitate description of the role of host-immune response in LHF. The STAT-1^−/−^ mouse model provides an accessible animal model for further study and the development of medical countermeasures targeting LHF.

## Data Availability

The original contributions presented in this study are included in the article. The raw data supporting the conclusions of this article will be made available by reasonable request directed to the corresponding author.

## Abbreviations

The following abbreviations are used in this manuscript:

LUJV	Lujo virus (*Mammarenavirus lujoense)*
LHF	Lujo hemorrhagic fever
STAT-1	Signal transducer and activator of transcription 1
LASV	Lassa virus (*Mammarenavirus lassaense*)
LF	Lassa Fever
LCMV	Lymphocytic choriomeningitis virus (*Mammarenavirus choriomeningitidis*)
MOPV	Mopeia virus (*Mammarenavirus mopeiaense*)
IPPV	Ippy virus (*Mammarenavirus ippyense*)
MOBV	Mobala virus (*Mammarenavirus praomydis*)
STAT-1^−/−^	STAT-1 constitutive knockout
IFrag^−/−^	Interferon alpha receptor and interferon gamma receptor double knockout
K2EDTA	Dipotassium ethylenediaminetetraacetic acid
RNA	Ribonucleic acid
qRT-PCR	Quantitative reverse transcriptase polymerase chain reaction
HC	Historical controls
BSL4	Biosafety level 4
DPI	Days post-inoculation
NHP	Non-human primate
